# Parental experiences transitioning neurological impaired young adults to adult services

**DOI:** 10.1016/j.hctj.2026.100139

**Published:** 2026-05-20

**Authors:** Hilary Noonan, Rajamanikandan Savarimalai, Pauline O’Reilly

**Affiliations:** a University Hospital Limerick, Limerick, Ireland; b School of Nursing and Midwifery, University of Limerick, Limerick, Ireland; c Health Research Institute, University of Limerick, Limerick, Ireland

**Keywords:** Parent's/caregivers experiences, Chronic Illness, Transitioning to adult services, Children with complex health care needs, Severe neurological impairment

## Abstract

**Background:**

Neonatal and paediatric care has been advancing. However, most young adults will need to transition from paediatric to adult healthcare services, which is more challenging and complex, especially for young adults with severe neurological impairment (SNI) due to significant healthcare needs. This study aimed to explore the experiences of parents of young adults with SNI who were part of the transition process of moving from children to adult healthcare services in an acute hospital setting.

**Method:**

An interpretative descriptive qualitative design was used to establish the experiences of eleven parents of young adults with SNI on transitioning from paediatric to adult healthcare services in an acute hospital setting.

**Results:**

Reflexive thematic analysis revealed a challenging and complex transition. Progress to adult healthcare services was a major shift in care. Additionally, challenges and worries about what is to come in relation to the transition and how to face the unknown situations. The parents of young adults with SNI involved in this study were not prepared or supported in moving from paediatric to adult healthcare services. This study provides in-depth insights into their transition experience.

**Conclusion:**

Findings from this study suggest that a more thoughtful and planned approach to transition is needed for young adults with SNI.

## 1 Introduction

Due to advances in neonatal and paediatric care, children with complex healthcare needs are living longer into adulthood. Consequently, most young adults will need to transition from paediatric to adult healthcare services, which is more challenging and complex, especially for young adults with severe neurological impairment (SNI). Many conditions in Ireland, such as diabetes and cystic fibrosis, have clear pathways for transitioning to adult services due to the existence of equivalent specialities in the adult healthcare service, which makes for a smoother process.^1^, ^2^ Although a national transition of care document from paediatric to adult services exists, there are currently no specific pathways for young adults with SNI.

In recent years, there has been a growing recognition of the link between the negative experiences aligned to moving from children to adult healthcare services and burnout in parents/caregivers, in addition to poor patient outcomes.^3^, ^4^, ^5^, ^6^, ^7^, ^8^ Parents of children with severe neurological impairment (SNI) face persistent and complex challenges. Their child’s condition does not improve, and in many cases, the future may involve declining health or increasing medical complexity. As a result, young adults with SNI continue to rely heavily on parental or caregiver support into adulthood. This sustained caregiving burden can lead to parental burnout, characterised by physical, emotional, and mental exhaustion resulting from prolonged stress. The transition to adult services represents a particularly stressful time, marked by uncertainty and concerns about continuity of care. Accordingly, the health and wellbeing of parents should be recognised as essential elements in the care of young adults with SNI.^4^

SNI has been defined as a term to describe disorders related to the central nervous system that can be either static or progressive and arise in childhood, resulting in motor and cognitive impairment as well as medical complexity.^9^ SNI is not a diagnosis. It is a phrase used to describe a group of patients with similar healthcare conditions, unique care needs, and challenges. Children with SNI have a wide variety of diagnoses, many of which are rare, and for some children, there is no underlying diagnosis. These children require high level of assistance with activities of daily living. They have significant healthcare needs, experience a high symptom burden and cannot self-report because they are non-verbal.^10^ Many children with SNI require the assistance of medical technology for feeding and breathing purposes and may also have seizures, dystonia, sleep difficulties, secretions, gut issues, constipation, contractures, and scoliosis. This group depend on parents/caregivers to continue as their main carers leading into adulthood. Children with SNI require family-centred care, and a comprehensive multidisciplinary approach from diagnosis, along the care trajectory and for some into adulthood.^11^, ^12^ Transitioning is a very stressful and challenging time, as their diagnoses are rare and unfamiliar to the adult healthcare teams. The adult healthcare team and general practitioner (GP) may lack expertise in these conditions.^13^

In Ireland, the age of eighteen represents the formal transition point at which paediatric services cease. Preparation for this transition within paediatric services is limited, often consisting only of referral letters from the paediatrician to adult speciality services and the general practitioner (GP). There is no equivalent adult service to oversee their complex care needs, and these young adults require input from multiple specialities, such as neurology, respiratory, gastroenterology, orthopaedics, and the possibility of palliative care. Therefore, paediatric services face significant challenges in facilitating effective transition. From the age of eighteen, paediatric services come to an end, regardless of their overall health, unless the young adult is at the end of life, which can make the transition appear abrupt for both families and professionals. As a result, when these young adults attend the emergency department (ED), they are treated under the adult team.^14^ Up to the age of eighteen, the paediatrician takes the lead in coordinating and managing care for children with SNI. Once the young adult with SNI turns eighteen, the GP is then expected to oversee and manage their ongoing care. They are not involved in the transition process other than receiving consultant letters. In Ireland, GPs act as central gatekeepers, providing private and state-funded care for children and adults.

Li et al. argue that the transitioning time should be arranged with flexibility and should not happen during times of a health crisis.^13^ In the literature, the age at which transition should occur is inconsistent.^15^, ^16^, ^17^ Parents play a crucial role in the care of their child with SNI, and no parent should experience unnecessary stress, burden, or fears going into adult services.^4^, ^18^, ^19^, ^20^

Despite the development of many guidelines and recommendations, service providers are still unsure of the best ways to prepare for and support the transition to adult services. There is a lack of evidence in relation to what happens to young adults with SNI and the experiences of parents after they leave paediatric care. Addressing this gap, the following study aimed to explore the experiences of parents of young adults with SNI with a focus on the transition process.

## 2 Research design

An interpretative descriptive design was used.^21^ This approach was chosen as it aligns with exploring important and complex subjective experiences of individuals with an aim of using the knowledge to influence healthcare practice and policy.

Ethical approval was attained for this study (Ref: HSEREC108/2023). As part of the ethics application, a data protection impact assessment and a research risk assessment were completed. To ensure rigour, the Standards for Reporting Qualitative Research (SRQR) guidelines were followed and attached in the supplementary file.^22^

### 2.1 Recruitment and procedure

A purposive sampling process was employed to source parents/caregivers who had experienced the process of their child/young adult transitioning to adult healthcare services. The inclusion criteria were parents of young adults with SNI aged between 18 and 21 years, who had already transitioned to adult services within the same acute hospital setting. Participants had transitioned from paediatric services between 11 months and 3 years. The young adults with SNI included conditions such as, cerebral palsy (GMFCS V), metabolic and undiagnosed conditions. In order to preserve participant anonymity and ensure confidentiality, no further identifying clinical detail are disclosed. Parents were excluded if their child was currently going through the transition process or if their child had died. A gatekeeper was used to identify suitable prospective participants. The gatekeeper selected individuals from previous clinic lists who had been under the paediatric service and were currently attending the adult service, aged between 18 and 21 years. Twelve parents were provided with study information, consent forms and contact details of the first author. On consenting to the study, parents/caregivers contacted the first author to arrange a suitable time and place for the interview. Both parents or one parent could participate in the interview; the choice was theirs.

### 2.2 Data collection methods

Data were collected using semi-structured interviews, which were guided by an interview schedule consisting of topics based on the findings from both clinical experience of the investigator working in paediatric neurodisability and the relevant evidence in the area based on the findings from the literature review^5^ (Table S1). A pilot interview was carried out with a general paediatric nurse to ensure that the interview schedule was clear and that it flowed in a logical sequence, along with effective probing strategies to collect informative original data to answer the research question.^23^, ^24^ Constructive criticism was taken on board following the pilot interview.^24^

The interviews took place between January 2024 and March 2024 and were carried out by the first author. Eleven interviews were face-to-face, and one was conducted on a General Data Protection Regulation (GDPR) compliant video conferencing software, viz. Microsoft Teams (MS Teams). All face-to-face interviews were held in a place decided on by the participants. Ten interviews took place in the participant’s home, one in the hospital setting and one on MS Teams. Nine interviews were with mothers, one was with a father, one with both parents, and one with a father and a family member. At the beginning of each interview, the interviewer established a rapport with the participant. Parents were asked to briefly introduce themselves and their adolescent. This helped them feel more at ease. Each participant was allowed to speak freely to expand on their points, even though an interview schedule was used to ensure that the research question, which aimed to explore the experiences of parents of the transition process involved in moving from children to adult services for their young adults with SNI, was addressed. This approach allowed for an in-depth portrayal of participants’ experiences. Following the interview, one parent decided to withdraw from the study. The first author complied with their wishes. Consequently, eleven participants remained in the study. Some participants were emotional during the interviews; however, they were not in any distress. A distress protocol was in place if required.

All interviews were audio recorded on a GDPR approved device. This allowed the interviewer to concentrate on the interview and listen attentively. Interviews were transcribed verbatim by the interviewer to ensure that participants' voices remained integral to their storytelling.^25^ Transcripts were produced by the HN and verified by PO’R. To ensure confidentiality, transcripts were pseudonymised with all identifying material removed.

### 2.3 Data analysis

This study followed Braun and Clarke’s (2021) six-phase process for conducting reflexive thematic analysis (RTA).^26^ The interview data were systematically analysed following the six steps: familiarisation with the data, generating initial codes, generating themes, reviewing potential themes, defining and naming themes, and producing the report. Reflexive thematic analysis was applied in a flexible manner to fit the data and research question, and helped provide a rich and detailed account of the data.^26^ In this study, thematic analysis and data collection happened concurrently.

As part of data analysis, the investigator became familiar with the content by reading and re-reading and making notes on initial observations. This was a time-consuming process that evolved, leading to new interpretations of the data going over and back between phases.^26^ However, this had huge benefits by allowing the first author to become immersed in the data. Gaps, pauses, and emotional tones were included in the transcription. Audio recordings were deleted following transcription. For this study, the methods of semantic and latent coding were used to ensure that no relevant excerpts were accidentally excluded.^26^ The investigator played an active role in interpreting codes and themes and identifying themes that were relevant to the research question. Finalising themes was a lengthy process, as all the interviews provided relevant, engaging, and varying points of view. Remaining critical of the data was important, which resulted in interesting themes being developed. To ensure rigour, the themes and subthemes were discussed and agreed upon with the other authors. In interpretive description, attention to rigour is extremely important to yield meaningful and useful results.^27^, ^28^ To provide a reliable description of the participant's experiences and prevent forming assumptions or incorrectly interpreting the data, the investigator engaged in reflexivity to increase the rigour of this study.^29^. To reduce the possibility of bias, the investigator has worked closely with her supervisor.

Data were reviewed so that the risk of patient identification was minimised. Extra caution was taken to ensure that no participants would be identifiable in any results published or during any presentation of this study.

## 3 Findings

The data were analysed to interpret the true meaning of parents' experiences after their child/young adult had transitioned to adult services for young adults with SNI. The analysis resulted in the development of three themes, namely, (1) Theme 1 Progressing to adult healthcare services – A shift in care, (2) Challenges and worries about what is to come, and (3) Facing the unknown. Each theme had two subthemes (Table 1). Together, these themes and subthemes provide a clear overview of the impact that moving from children to adult healthcare services had on parents and their families. Quotes from the interviews will be used to illustrate the themes.

**Table 1:** Themes and sub-themes.

No	Theme	Sub-theme
1	Progressing to adult healthcare services – A shift in care	➣ Abrupt change – no preparation ➣ Sense of Loss
2	Challenges and worries about what is to come	➣ Wakeup call ➣ The General Practitioner’s (GPs) role in facilitating the transition
3	Facing the unknown	➣ Ongoing care ➣ Stress in daily life

### 3.1 Theme 1: Progressing to adult healthcare services – a shift in care

This theme focused on the time when parents first heard that their child/young adult was going to be transferred from children to adult healthcare services. This process was carried out in an ad hoc way; sometimes at a follow-up appointment or as an inpatient when their young adult was nearing 18 years of age. Parents had no notification and were shocked. Parents highlighted that this process was not managed well. This abrupt change with no preparation and not knowing whom to turn to was what the participants placed considerable emphasis on during the interviews.

#### 3.1.1 Abrupt change – no preparation

Eight parents reported that they first heard that their child was moving to the adult service from their consultant paediatrician while their child was still an inpatient or attending a clinic at the age of 17 years. They were informed that if they presented to the hospital at 18 years old, they would be cared for in an adult healthcare setting. One parent discovered it from a medical student, and another from a parent’s forum. Another parent was informed that their consultant could only see them while their child was in school, and there was no further discussion in relation to the matter. One parent never had the chance to discuss the transition with their paediatrician.

*“I know we had no choice due to age…the communication piece is missing…that's where you just feel isolated and lost. You’re lost in the system. You're absolutely lost in the system”* (M+FF09a).

Four of the eleven parents were linked to the nurse coordinator for life limiting conditions due to a decline in their child’s health status. They outlined that they received more information from her on what to expect in the adult service. Parents were asked if they felt involved in the conversation about moving from children to adult healthcare services. One parent did feel involved and attended a multidisciplinary team meeting where the parent requested to stay in children services. This parent reported they were given no choice at the end of it. The other parents were informed that letters would be sent to each of the adult specialists. Thereafter, they would be put on an outpatient waiting list, but they were not sure which specialist consultant would see them. They had no link to an adult specialist nurse until they were reviewed by the consultant. Ten parents said they were not involved in the discussion and had no preparation. They would have liked more information, as they had no idea what to anticipate and were confused. They were given no guidance and had no one to contact for advice.

*“But we have no choice. I know that we have no choice because of the age….you're trusting your child to the care of people that you don't know them at all”* (M+Ms05a).

#### 3.1.2 Sense of loss

Within the children’s healthcare services, the nurse coordinator for life-limiting conditions had been involved with four young adults and was the parents point of contact. She was familiar with their child’s condition, medical team, and additional specialities involved. There is no equivalent nursing post in the adult healthcare services to continue the work.

*“Just having that constant in your life is important….so that’s a huge loss….familiar face… like somebody kind of navigating. I know we’ve lost a lot of our connections…nurse….dietitian…she had nobody to refer us to”* (M+FF01aa).

All parents spoke about the important link that they all had with a dietitian with whom they liaised and were in regular contact with for years. They relied on this service and never expected it would suddenly be gone when their child reached the age of 18. The parents reported feeling isolated in the adult services, as there was no designated link person to oversee their child’s care with them. They no longer received regular dietitian follow-up or consultant reviews. All parents expressed the need for a nurse coordinator and a continued dietetic service who knows their child and how to support them in adult services. Parents felt isolated and anonymous within the adult healthcare service.

*“In adult ____ he always gets great care inside. But when he comes out, it's like as if the hospital doesn't exist anymore. There is no one there. There are no appointments. There's nothing happening, OK”* (M+FF09a).

### 3.2 Theme 2: Challenges and worries about what is to come

This theme explored how parents worried about meeting many different professionals and having no one team or designated nurse coordinator to oversee their child’s care. Parents highlighted the challenges in the adult emergency department (ED) and adult wards and the role of their general practitioner. They were unsure how the system worked compared to the paediatric service.

#### 3.2.1 Wakeup call

In the paediatric services, parents were aware of how the ED worked. They presented and remained under the same named paediatric consultant. Parents did not feel the responsibility of having to remember everything. All parents emphasised that their child continued to have the same needs, and all that had changed was their biological age. They were not going to suddenly improve. They continued to require someone to always take care of them. Each time the young adult presented to the adult ED, their care was overseen by the on-call team, a different medical team each time. The time spent in the ED was very stressful for parents and their children, as they were now among the other adults on trolleys in the corridors.

*“Ohh, it was horrendous, we were in the middle of like everybody….now the staff, they were very good… they’re under awful pressure as well”. “I was sitting on the chair and I didn’t get a blanket or a pillow or anything like that”* (M+FF02a).

In the children healthcare services, all parents reported that they had trust and a great rapport with their paediatric team. They were always prioritised to get a single room. However, in adult services, parents state that for each admission, you are under a different medical team. They felt overwhelmed and frustrated with the number of people involved. They felt a huge responsibility for having to remember everything about the care needs of their child and they were required to impart this information multiple times to different members of the medical team. They did not always have a medical chart. They stated they felt anonymous, and the personal touch and compassion were no longer evident.

*“There’s no continuity. No time to build a rapport or trust. He whisked in and whisked out, It lacked, I suppose, compassion…. …you’re anonymous, you’re a number…. They left us home…they left everything up to myself”* (M+FF02a).

On admission, the young adult with SNI didn’t always get a single room. Their adult children were in big wards, with beds divided only by curtains. There were no facilities to weigh them or change them. One parent reported that there were no slings for the hoist. Another reported that they were expected to do all the care by themselves.

*“Equipment is massively lacking….no weighing scales for wheelchair person…Ridiculous…no wipes, nappies. I’ve always had to bring everything with me”* (M+FF12a).

One parent recounted that their young adult had an unnecessary admission. His feeding tube had dislodged from his stomach and fallen out. As the staff were unable to locate another tube, he had to be admitted. Although the parents were allowed to stay, they were only offered a chair, as there were no facilities for parents/caregivers. Their young adult was only treated for the issue that they presented with and discharged back under the care of their general practitioner (GP), with no regular follow-up appointments.

#### 3.2.2 The general practitioner’s (GPs) role in facilitating the transition

All parents were familiar with their GP. However, six parents found it very difficult to get an appointment with their GP and had no meaningful relationship with their GP. They did say that they were good for general matters. Parents were unsure if the GP was able to take over the medical coordination of their child’s care. The parents outlined that the GPs were already overworked, were unsure of their child’s condition, and had no connection to the hospital other than written correspondence.

*“Our GP …. you feel that responsibility to telling him nearly how to manage us or what to do. All of her prescriptions have come from the hospital … getting a prescription amended or I don't know if the GP will be happy to do that”* (M+FF06a).

All parents stated they had to go straight to the hospital if their child was unwell. Five parents highlighted that they had a good relationship with their GP and agreed they should be involved more, like attending an online meeting with the paediatric consultant to gain a greater understanding before the transition occurred. Two parents commented that the GP supports them in maintaining their own health. However, three GPs were nearing retirement, and the parents were apprehensive about meeting new GPs. One parent was concerned about having to guide the GP and worried if the GP would be open to making any adjustments.

*“Yeah, but I think he needed for the normal things. Yes, they can sort it out; they sent the letter. Ohh, for all his medical issues, the GP tells me it’s not his area, he knows nothing about my son’s complex needs”* (F+FF03a).

### 3.3 Theme 3: Facing the unknown

The third theme identified that all parents worried about the future due to the lack of continuity of care and the lack of regular hospital follow-up appointments. Parents emphasised that turning eighteen years of age affected all services, which added to their already stressful lives. They discussed their personal health, their other children, their worries and fears for the future and made suggestions for improvements.

#### 3.3.1 Ongoing care

All parents worried about not having reviews while their young adult was well. Having no direct link to a healthcare professional for guidance and no access to a dietitian unless they presented to the hospital was very troubling. They stated they are doing what they learned themselves from the paediatric service and hoped that they were getting it right. Two families were still on the waiting list for their first review by an adult specialist consultant. Therefore, the adult specialist nurse was not available to them. One parent was informed she would see a specialist consultant as an inpatient in the adult service, but it never happened. The consultant did phone her after their discharge, but it was of little benefit as she wasn’t prepared for the call.

*“We have developed those relationships over the course of her[child’s] life and now what? You know it is like it's a burden it's becoming and I don't want to talk about my child as if she's a burden because she's not a burden”. I felt it [care] was more shared in the paediatric service and I felt we were heard”* (M+Ms05a).

One father said that his GP is helpful in making referrals but felt his son’s condition is worsening while waiting for appointments. Parents stated that their child requires more care as their needs are changing. They have no one to contact who understands their total healthcare needs. Only one mother reported meeting an adult specialist consultant while an inpatient under paediatric care, which was not planned. She said it was different and felt the adult consultant was out of his depth. However, as she now had a link to the adult nurse specialist for that team, this helped her move into adult services for that part of her son’s care. All parents stated that the negative media coverage on ED waiting times has played a factor in them delaying their presentation to the adult ED.

*“A 2 year old in an adult's body….it's not terribly structured now …a big wakeup call… So there is no kind of proactive thought into her care. Why is everything having to be presented to the emergency department in order to get to see who you need to see”* (M+FF06a).

#### 3.3.2 Stress in daily life

Parents spoke about the start of their child’s journey, the feeling of isolation, and felt it was happening again. All participants highlighted the challenges of going to adult healthcare services. A recurrent issue that was highlighted was the need for information, the lack of communication and continuity of care. Not knowing the teams and having no direct point of contact was problematic.

*“Accepting _______ at the start was very difficult….isolated……and it brought us to the depths of despair and back again, and here we go again”* (M+FF04a).

When their child presented to the ED or was admitted to big open wards, they described how this impacted their own health and wellbeing. It was stressful. All parents highlighted that their child turning 18 years of age had a knock-on effect on all services. This magnified their difficulties in looking after themselves. They were unable to make or attend appointments for themselves. Parents were getting older and were exhausted, feared for the future of their young adults, and spoke about the impact on siblings.

*“I can't plan to have my surgery, you just feel alone, feel like I’m in a prison. I feel a really big gap. I feel like my battery is very low”* (M+FF08a).

All parents recognised the inadequacy of the transition process from children to adult healthcare services. They made suggestions for improvement in the transition process.

*“A sit-down actual plan…a hospital passport that could go with the child. I think a coordinator will be invaluable and that role created in adults where they're the connector”* (F+FM11a).

## 4 Discussion

This study has explored parents’ lived experiences of their child/young adult with SNI transition from children to adult healthcare services. Parents experiences have highlighted the practical and emotional distress they encountered and provide new insights into what is required when moving from children to adult healthcare services. The main findings across all the themes were that planning was inadequate in the transition between children’s and adult services. In line with the evidence, some condition-specific paediatric illnesses have transition pathways where services are equivalent to those of adult teams.^1^, ^2^ However, for young adults with SNI, there was no clear transition pathway.

### 4.1 Managing the transition process

In this study, parents learned that their child/young adult was going to adult services primarily from their consultant, but this information was inconsistent, unplanned, and provided at a late stage. These were not planned discussions. The only communication was referral letters written to different adult specialists’ teams, as there was no equivalent adult team to oversee holistic care. Parents were given no choice once their child turned 18 years old. The literature itself is inconsistent regarding the ideal age for healthcare transition.^15^, ^16^, ^17^ Parents concern was that their children were now treated as adults due to their biological age, despite their neurological impairment. Participating parents, being experts in their child's care and advocacy, wanted to be involved in the transition process.^5^ The evidence consistently shows that parents require support to navigate these services.^4^, ^5^, ^10^, ^12^, ^30^, ^31^, ^32^

In Ireland, there is widespread recognition of the necessity of implementing healthcare transition services for young adults with long-term conditions, as well as those with SNI.^12^, ^18^, ^31^, ^32^, ^33^, ^34^ Despite this, the parents of young adults with SNI involved in this study were not prepared or supported in moving from children to adult healthcare services.^35^, ^36^, ^37^

### 4.2 Reality of the adult service

Parents described both practical and emotional challenges. Practically, parents described their experiences of being in the ED and the admission of their child to an adult ward. There were no facilities to weigh or change their child/young adult, only a curtain separating them from the adjacent patient. They were worried about the length of time spent in the ED. One parent, having brought her own hoist slings, discovered that they were incompatible with the hospital hoist, and distressingly, the hospital slings could not be found. One young adult presented to the ED as his peg tube fell out and was admitted unnecessarily due to having no peg tube available in the hospital. They were not always given a single room or cubicle. Parents were not offered breaks, a blanket or a pillow. They sat on chairs. Although this is new to these participants in this adult healthcare setting, the organisation's environment needs to be reviewed.

### 4.3 Impact on young adults and families

Emotionally, parents expressed strong feelings of being frustrated and overwhelmed by the number of adult teams they met at each admission. Parents felt isolated, alone, and with the lack of communication, they had no trust or rapport with the adult team members. They felt an overwhelming responsibility for ensuring that all teams had all the information. These feelings are acknowledged by Bratt et al. and Brown et al*.* studies that highlight parents frequently experience a sense of loss, fear, and uncertainty after leaving their trusted paediatric teams.^38^, ^39^ Parents stated their child was treated for what they presented with and discharged back to their GP. When they left the hospital, they had no one to contact or turn to, and there were no regular reviews. They felt anonymous and worried. Lack of communication and poor follow-up post transition were classified as major issues and barriers.^2^, ^14^, ^16^, ^40^, ^41^, ^42^ The evidence does highlight that care is disjointed.^12^, ^43^

### 4.4 Lack of coordination

Consistent with prior research, parents in this study expressed the need for a nurse coordinator to oversee their child's/young adult's care with them. This is a nurse who is familiar with their child's specific care needs, as they do not want to take on this additional coordination responsibility themselves.^5^, ^12^, ^30^, ^31^, ^32^, ^44^ An important and novel finding is that all parents were upset that the outpatient dietetic service just stopped, as there was no one in the adult service to hand over to. One parent described his son’s health as worsening due to having no one to contact and no one understanding his total healthcare needs. Parents stated they are doing what they learned in paediatrics. The evidence highlights the significance of transition planning and supports the necessity for a coordinator role, for ensuring positive long-term clinical outcomes.^18^

### 4.5 The GP role in facilitating transition

There was much discussion regarding whether their GPs were able to effectively coordinate care for their young adults with high medical needs. All participants agreed it was not appropriate since, as children, if they became unwell, they needed to go straight to the hospital, where all care was overseen by the paediatric service. This is consistent with the literature, which noted parental lack of confidence and GPs being "out of the loop", particularly when they did not regularly see the child while under a paediatrician's care.^45^, ^46^ Parents struggled to see how already overworked GPs could help with the hospital aspect of care. Nonetheless, this study revealed a strong consensus among parents who still saw GP involvement as crucial for addressing general matters. Despite evidence suggesting early GP engagement improves transition outcomes for conditions like cerebral palsy,^47^ current literature offers little guidance on how GPs can effectively coordinate care for young adults with significant healthcare needs.^2^, ^45^

### 4.6 Ongoing care needs

The practical and emotional challenges of navigating the newfound change affected parents’ health. An 18th birthday for their young adult was very difficult. They do not suddenly improve or become somewhat independent. With the abrupt stoppage of children’s services at 18 years of age, they faced immense challenges as their young adults with complex needs, who remain fully dependent and cared for at home, require increasing care into adulthood as their healthcare needs change/decline. This sudden change left parents fearful about the future, deeply stressed about ongoing care, and too exhausted to schedule appointments for themselves or engage in self-care, consistent with earlier research.^35^, ^36^, ^37^ While much research on parental well-being focuses on mothers,^48^, ^49^ recent studies are exploring fathers' experiences, suggesting potential differences within the same family unit.^50^, ^51^ Participants in this study were predominantly mothers, reflecting a common bias in research on parental experiences.^48^ Nevertheless, both mothers and fathers expressed similar concerns regarding the transition process.

### 4.7 Every day stress

This study highlighted parents' enduring memories of their child's initial diagnosis and the profound impact their young adult with SNI had on their other children. They tried to maintain everything normal in their lives by keeping them involved in activities with their friends. They didn’t want them feeling responsible for their sibling’s care.^52^, ^53^, ^54^ According to a study by Allen et al.,^9^ siblings normalise their experiences. Depending on their age and development, they worried about their parents and reported them as being busy, which affected the time they had available.^9^ It is important to understand the effects that caring for a young adult with SNI has on the entire family. Hence, parental health is important for sustaining long-term care for their child well into adulthood.^9^, ^12^, ^31^, ^32^ Previous research has shown that when parents are healthy, they are more capable of supporting their child/young adult.^4^, ^35^

### 4.8 Clinical and research implications

This research significantly expands the understanding of how poor transition planning impacts parents of young adults with SNI. The findings emphasise the critical need for paediatric services to develop a robust, well-planned transition pathway that will ultimately improve outcomes for this vulnerable population. After changes have been implemented within the service, it is important to explore the barriers and facilitators of those changes from the viewpoints of parents and professionals who are part of the transition.

### 4.9 Limitations

Though this study provides in-depth insights into their transition experience, all participants were from the same healthcare setting. However, selection bias was avoided as the first author was unaware of participant identities. A purposive sampling process was employed to recruit parents/caregivers with relevant experience. This was guided by predefined inclusion criteria established prior to recruitment. Data collection continued until saturation was reached, with no new themes emerging. The investigator applied reflexivity to identify and manage potential bias. The results of this study cannot be generalised because of the smaller sample size, which is typical of qualitative research. Despite this limitation, the findings have contributed to expanding current knowledge and understanding of why a planned transition process is necessary.

## 5 Conclusion

These parents of young adults with SNI faced huge challenges navigating the transition to adult services on their own. These included environmental barriers, uncoordinated support, the absence of a dedicated adult neurodisability team, and the fact that there is only a dietetic service for inpatients. Although parents are experts in caring for and advocating for their child’s care needs, they are not medical experts. This study clearly shows that parents received no preparation for their young adults with SNI transitioning to adult healthcare services. Parents in this study were left feeling unprepared, uncertain of available supports, and isolated by the abrupt transition, with no one to contact in the adult healthcare setting. Evidence strongly suggests that a more thoughtful and planned approach to transition can lead to much more successful outcomes. Parents in this study offered recommendations they believe will be beneficial to the transition planning process.

This research significantly expands the understanding of how poor transition planning impacts parents of young adults with SNI. The findings emphasise the critical need for paediatric services to develop a robust, thoughtfully planned transition pathway, which will ultimately improve outcomes for this vulnerable population.

## Ethical approval and informed consent statements

The study received ethical approval from the relevant ethics committee (Ref: HSEREC108/2023). Participants provided informed consent including consent for publication.

## Funding

The author(s) received no financial support for the research, authorship, and/or publication of this article.

## Declaration of Competing Interest

The authors declare the following financial interests/personal relationships which may be considered as potential competing interests. Hilary Noonan reports no financial support was provided by University Hospital Limerick. Hilary Noonan reports a relationship with University Hospital Limerick that includes: employment. - If there are other authors, they declare that they have no known competing financial interests or personal relationships that could have appeared to influence the work reported in this paper.

## Data Availability

The authors do not have permission to share data.
