# Supplementary material for: Parental experiences transitioning neurological impaired young adults to adult services
Source: Health Care Transit. 2026 May 20;4:100139. doi: 10.1016/j.hctj.2026.100139 (PMC13213692; doi:10.1016/j.hctj.2026.100139)
Supplement: Supplementary file 1 — Supplementary material [file mmc1.docx]

**Table S1: Semi-Structured Interview Schedule**

| **Topic** | **Probe** |
| --- | --- |
| **Building rapport** | |
| - Tell me about yourself and your son or daughter | - How are your children doing? |
| **Paediatric relationship – continuity of care** | |
| - How would you describe your relationship with your child's paediatric team? | - Who were you involved with on the team? |
| **Transition process** | |
| - When did you first learn about the transition to adult services? - What was your initial understanding of the transition process? - How did you feel about the transition, and were there any concerns? | - What age was he/she? Where did this happen, in a clinic, ward, etc? - How were you told? - What did that mean for you? How did you think it was going to happen? |
| **Lack of Communication & Coordination** | |
| - In what ways did you feel involved or listened to during this process? | - When was this? |
| **Coordination with healthcare facilities** | |
| - What aspects of the transition worked well, and what challenges did you face? - What improvements would you suggest for the transition process? | - How did it make you feel? - Did you feel supported? |
| **Emergency Department** | |
| - When and where did you first interact with the adult care team? | - Can you remember who took the lead? |
| **Adult health service abrupt change, stress, and isolation** | |
| - How well do you feel the new team understands your child's condition and medical needs? | - Can you elaborate? - What was it like meeting them for the first time? |
| **GP Role** | |
| - How well does your GP understand your child's condition? - What are your thoughts on your GP's involvement in the transition process? | - Did they have the chart? Did you have any letters? - Did you see your GP much? Do you have a good relationship? - Does he/she understand who you are involved with? |
| **Parental health** | |
| - What do you do to support yourself through this process? - Do you have any other questions or comments for me today? | - In what way? - Do you have much support? - Would you like to add anything further? |
